# Psychological distress, psychosocial factors, and physical inactivity among older women and men in Sweden: a population-based study

**DOI:** 10.1186/s12889-025-24868-6

**Published:** 2025-10-22

**Authors:** Susanne Nygård, Sanna Tiikkaja, Lena Lönnberg, Johnny Pellas, Michail Tonkonogi, Maria Liljeroos, Marina Arkkukangas

**Affiliations:** 1 Department of Medicine, School of Health and Welfare, Dalarna University, Falun, Sweden; 2 Department of Public Health and Caring Sciences, Social Medicine/CHAP, Uppsala University, Uppsala, Sweden; 3 Centre of Clinical Research Sörmland, Uppsala University, Eskilstuna, Sweden; 4 Department of Public Health and Caring Sciences, Uppsala University, Uppsala, Sweden; 5 Centre for Clinical Research Västmanland, Uppsala University, Västerås, Sweden; 6 Department of Health, Medicine and Caring Sciences, Linköping University, Linköping, Sweden; 7 Department of Physiotherapy, School of Health, Care and Social Welfare, Mälardalen University, Västerås, Sweden

**Keywords:** Exercise, Kessler-6, Mental health, Physical activity, Social participation

## Abstract

**Background:**

Physical inactivity is a major public health concern worldwide. Psychological distress is linked to physical inactivity, which increases the risk of several diseases. Women tend to be more physically inactive than men. Moreover, physical inactivity increases with age in both sexes. Therefore, this study aimed to investigate the association between psychological distress and physical inactivity in adults aged ≥ 65 years, the role of psychosocial factors and explore sex-based differences.

**Methods:**

This study included 14,213 older adults, comprising 7,069 women (52%) (median age = 75 years), who responded to a survey questionnaire sent to a random population sample in Mid-Sweden in 2022. The response rate in the population aged ≥ 65 years was 61%. The association between psychological distress as defined by the Kessler-6 and physical inactivity (< 150 min/week of physical activity) was analyzed using binary logistic regression, adjusting for sex, age, country of birth, educational level, and psychosocial factors.

**Results:**

Overall, 30% of the participants reported psychological distress, and 45% were physically inactive. Psychological distress and physical inactivity were significantly associated (*p* < 0.001), being more common among women (*p* < 0.001). In the fully adjusted model, the Odds Ratio (OR) for physical inactivity was 1.46 (95% confidence interval (CI) 1.43–1.50) for women and 1.70 (95% CI: 1.65–1.74) for men regarding moderate psychological distress and 2.87 (95% CI: 2.72–3.04) for women and 2.43 (95% CI: 2.28–2.58) for men regarding serious psychological distress. Not participating in social activities in the last 12 months was associated with physical inactivity in both women and men.

**Conclusions:**

Psychological distress is significantly associated with physical inactivity among older adults. Participation in social activities was identified as an essential factor in addressing physical inactivity. Social connections and physical inactivity are important factors to consider when supporting older adults’ mental health. Public health interventions should promote and raise awareness of physical and mental health as well as the social dimensions of aging, while also considering age and sex-based differences.

## Background

Physical inactivity is a major global public health concern. Recent data indicate an increase in physical inactivity among adults [1]. According to the World Health Organization (WHO), nearly one-third of all adults worldwide are physically inactive [2] a fact supported by previous research [1].

Globally, women tend to be more physically inactive than men but with increasing age, physical inactivity increases for both men and women [1, 2]. In Sweden, some studies have shown that older women and men are equally inactive, with inactivity increasing with increasing age [3]. However, further research has indicated that older men are more physically inactive than older women, and that women are significantly more likely to meet the recommendations for physical activity [4].

In this study, in accordance with global recommendations on physical activity, we define physical inactivity as < 150 min/week of moderately intense activity [5, 6]. Current recommendations for adults aged ≥ 65 years include engaging in 150 min per week moderately intense physical activity, muscle strengthening activities two times per week, and performing multicomponent physical activity to prevent falls and fall-related injuries, and for significant health benefits [3, 6].

Previous research has shown that physical inactivity increases the risk of common diseases such as cardiovascular disease, type 2 diabetes, cancer, and obesity, as well as depression, neurocognitive disorders, and premature death [3, 7]. The relationship between physical inactivity and mental illness is well documented [7, 8]. Studies have indicated that being physically active can reduce the risk of mental illness, whereas those with mental illness are at higher risk of being physically inactive [6–8].

Mental illness is an overarching term for diverse conditions of varying severity and duration, including psychological distress and psychiatric disorders [9–11]. Psychological distress refers to normal reactions to stressful situations that persist for a limited time. These reactions include symptoms such as low moods, anxiety, and concentration problems. However, they have a lower impact on overall functioning than psychiatric disorders. Sometimes psychological distress can develop into psychiatric disorders such as depression or anxiety [9–11]. This study investigated psychological distress.

Over the years and with life experiences, many older individuals have developed effective strategies to manage psychological distress. However, aging is associated with reduced psychological flexibility and resilience, making older adults vulnerable to the consequences of prolonged stress and negative life events [12]. Women are particularly vulnerable to factors, such as living alone and being widowed, which increase their risk of psychological distress [13]. In a previous study of older adults, women reported higher levels of psychological distress than men and distress was strongly associated with worse social functioning in both sexes [14]. This aligns with research indicating that psychological distress impact adults in various ways, including their social lives, relationships with family and friends, and ability to carry out everyday tasks [15]. Individuals with moderate psychological distress had somewhat reduced functions, and those with serious psychological distress had greatly reduced functions in these areas. Therefore it is important to consider the severity of psychological distress [15].

Common factors associated with mental illnesse that increase with age include reduced functional capacity, fewer social contacts, and deteriorating physical health [8, 16]. Many older adults who report poor mental health also have poor physical health [8]. Therefore, physical activity promotes healthy ageing [6]. Previous studies have shown that physical activity has a protective effect on both prevalent and incident depression [17] and reduces the prevalence of mental disorders, especially depression and anxiety [18]. Furthermore, older adults engaging in physical activity has the potential to mitigate depression and depressive symptoms [19]. In addition, physical activity positively affects several age-related diseases in older adults [20], and improves and maintains functional ability [21]. It is therefore recommended that older adults follow the recommendations for physical activity of at least 150 min per week as well as muscle strengthening activities and multicomponent physical activity [3, 6, 20]. However, even at lower levels of physical activity, health benefits were generally observed in this age group [22].

Thus, as mental illness is common among older adults [8, 16] and physical inactivity is increasing [1] it is of great interest to study the possible associations between mental illness and other factors that may influence this association. One model, well-established in public health, is the Rainbow Model serving as a way to describe the factors that influence health and its distribution in the population, and encompasses both individual and contextual factors [23, 24]. The choice of covariates in this study was based on this model.

Furthermore, the well-known differences in physical activity habits between men and women [1, 6] highlight the need to explore how mental illness is related to physical inactivity. Therefore, this study aimed to investigate the association between psychological distress and physical inactivity in adults aged ≥ 65 years, the role of psychosocial factors and explore sex-based differences.

## Methods

### Design and setting of the study

This cross-sectional study is based on data from the Life and Health Surveys, which were collected in the spring of 2022 (LH 2022) in five regions of Mid-Sweden (Uppsala, Värmland, Västmanland, Sörmland and Örebro) in collaboration with Statistics Sweden.

The questionnaire was both paper-based and online and was distributed to 78,117 persons aged 18 years or older in the five counties [25]. Statistics Sweden used the Swedish Total Population Register for the sampling frame, and a stratified (age-group, sex and municipality/county) random sample was drawn. These represent the population of 1,2 million people living in 55 municipalities. The total response rate was 45% and similar for the five counties, ranging from 43% to 47%. The distribution was discontinued after three reminders were sent via SMS to those with a registered mobile phone number. For other respondents, data collection was discontinued after two postal reminders. The survey included questions on health, living conditions, and health-related behaviors. Data on sex, age, country of birth and educational level were retrieved from the population registers hosted by Statistics Sweden. The data from the registries and questionnaires were merged and pseudonymized before delivery for further processing in the regions of Mid-Sweden. Descriptions and descriptive analyses of the data are presented in Swedish public health reports [26, 27].

The definition of an older person varies in the literature, ranging from 60 years of age, according to the WHO, to 65 years of age, according to the United Nations [28]. We chose the latter because 65 is the general retirement age in Sweden. According to Statistics Sweden, nearly 346,000 persons in the selected age group lived in Mid-Sweden by 2022. The response rate for the population aged ≥ 65 years was 61%, comprising 16,142 individuals who responded to the survey. A total of 1,929 individuals were excluded from this study since they had missing data. They were older (median = 79 years), had lower level of education (11% college/university) compared to the included persons [29]. Sex distribution was similar. Most of the excluded individuals lacked data on key covariates, psychological distress (*n* = 1,203) and physical inactivity (*n* = 350). In all 14,213 older adults were included in the study.

### Dependent variable – physical inactivity

Physical inactivity was measured with two questions: “During a regular week, how much time do you spend exercising on a level that makes you short winded - for example running, fitness class, or ball games?” (0 min/no time, < 30 min, 30–59 min, 60–89 min, 90–119 min, and ≥ 2 h) and “During a regular week how much time are you physically active in ways that are not exercise - for example walks, bicycling, or gardening?” (0 min/no time, 30 − 59 min, 60–89 min, 90–149 min, 150 − 299 min, and ≥ 5 h). These two questions were recommended and validated by the Swedish School of Sports and Health Sciences for measuring physical activity. The sum of activity minutes calculated from the two questions consists of the exercise time multiplied by two added to the total time spent on everyday physical activities [30]. In this study, the respondents were considered physically inactive if they did not meet the WHO recommendation of ≥ 150 min of physical activity per week [6]. The distribution of physical activity was divided into two categories: physically inactive = < 150 min/ week or physically active ≥ 150 min/week.

### Independent variable - psychological distress

Psychological distress was measured using the Kessler-6 (K6). The K6 is a brief screening tool widely used to investigate psychological distress in surveys and clinical settings. The K6 consists of six questions that require respondents to consider their experiences with the following symptoms over the last 30 days: feeling nervous, hopeless, restless or fidgety, depressed, and feeling that everything was an effort and worthless [31]. The psychometric properties of K6 have been demonstrated in adults in general [32] and older adults in particular [33]. A recent study using the Swedish version of the K6 demonstrated satisfactory construct validity [34].

For each question, a score of 0, 1, 2, 3, or 4 was assigned to the answer: “none of the time,” “a little of the time,” “some of the time,” “most of the time,” or “all of the time,” respectively. The responses to the six items were summed to yield a K6 score of 0–24. Respondents scoring < 5, ≥ 5 to < 13, and ≥ 13 were categorized as having no/low, moderate, and serious psychological distress, respectively [15].

### Covariates

Background covariates included sex, age, country of birth, and educational level, these were considered as potential confounders. Age was categorized into the following groups: ‘65–74’, ‘75–84’, and ‘85–102’. Sex was defined as ‘woman’ and ‘man.’ Country of birth was categorized into ‘born in Sweden,’ ‘born in another Nordic country,’ and ‘born in the rest of the world.’ The highest achieved level of education was defined as ‘elementary school’, ‘high school’, or ‘college/university’.

Psychosocial covariates included, living with a spouse/partner, participation in social activities in the last 12 months, having trust in other people and having a friend to confide in. Respondents that answered that they live most of the time during the week together with a ‘spouse/partner’ were categorized as living with a spouse/partner, while answering ‘no one,’ parents/siblings’, ‘children ˂18 years’, ‘children ˃18 years’ or ‘other adults’ were categorized as not living with a spouse/partner. Participation in social activities was measured by the question “During the last 12 months, have you regularly engaged in activities with others – for example sports, music/theatre, studies, religious meetings, choir singing, political association, or other association activities?” (‘yes’/’no’). Trust in people was measured with the question, “Do you think that you can trust most people in general?” (‘yes’/’no’). Having a friend to confide in was assessed by the question “Do you have someone you can share your innermost feelings with and confide in?” (yes/no).

### Statistical analysis

First, we used chi-square statistics to evaluate whether there is an association between psychological distress and physical inactivity. Second, descriptive analysis was conducted to show the distribution (n and %) of the study population for the included covariates according to sex and physical inactivity (‘yes”/’no’). Differences in the prevalence of psychological distress and physical inactivity according to sex were assessed using chi-square tests. Finally, binary logistic regression was performed, displaying ORs with 95% CIs and corresponding p-values for the covariates in the respective model. The models show the association (OR) between psychological distress (independent variable) and physical inactivity (dependent variable); the first model shows the crude (unadjusted) OR. Step 1 was adjusted for background covariates (age, country of birth, educational level). Step 2 included additional adjustments for psychosocial covariates (living with a spouse/partner, participating in social activities in the last 12 months, having trust in other people, having a friend to confide in). These analyses were conducted for the total sample as well as for women and men. Nagelkerke’s test was used to describe the proportion of explained variance. We used a calibration weight created by Statistics Sweden by using the following covariates, sex, age, level of education, country of birth and geographic area from Swedish registries, for calculation of proportions and logistic regressions, to reduce bias due to non-response or selection [25]. All statistical analyses were performed using SPSS version 29, with *p*-values ˂0.05 considered statistically significant.

## Results

In all, 26% of the participants reported moderate and 4% reported serious psychological distress, and 45% were physically inactive (Table 1). A statistically significant association was found between psychological distress and physical inactivity (*p* = < 0.001). A higher proportion of women than men were physically inactive, 49% and 41% respectively. Additionally, moderate and serious psychological distress were more common among women (30% and 5%, respectively) than among men (23% and 4%, respectively), with the differences significant (*p* = < 0.001) (Table 1).

**Table 1:** Distribution of psychological distress and physical inactivity on the total study population and by sex

		Total *n* = 14,213 (%)	Women *n* = 7069 (%) (52)	Men *n* = 7144 (%) (48)
Psychological distress*	No/low	9886 (69)	4630 (65)	5256 (74)
	Moderate	3728 (26)	2076 (30)	1652 (23)
	Serious	599 (4)	363 (5)	236 (4)
Physical inactivity*	Yes	6589 (45)	3459 (49)	3130 (41)
	No	7624 (55)	3610 (52)	4014 (59)

The proportions are based on weighted data

Physical inactivity: yes = ≤ 0–149 min/week, no = ≥ 150 min/week

*P < 0.001

Physically inactive participants reported more moderate psychological distress (32%) than participants who were physically active (22%) (Table 2). Age and educational level were distributed differently among those who were physically inactive; the former group was older and had a lower level of education. Country of birth was similarly distributed in both groups, with the vast majority being born in Sweden. Psychosocial covariates were also differently distributed: the proportion of participants with lower social activity participation was higher among those who were physically inactive than among those who were physically active (71% vs. 49%). The proportion of those not living with a spouse or partner, not having trust in other people and not having a friend to confide in was also lower among those who were physically inactive.

**Table 2:** Distribution of physical inactivity and covariates for the total study population and by sex

		Total *n* = 14,213(%)		Women *n* = 7069(%)		Men *n* = 7144(%)	
		Physically inactive *n* = 6589 (45)	Physically active *n* = 7624 (55)	Physically inactive *n* = 3459 (49)	Physically active *n* = 3610 (52)	Physically inactive *n* = 3130 (41)	Physically active *n* = 4014 (59)
		n (%)	>n (%)	n (%)	n (%)	n (%)	n (%)
Psychological distress*	No/low	4070 (61)	5816 (76)	2031 (58)	2599 (72)	2039 (65)	3217 (80)
	Moderate	2084 (32)	1644 (22)	1158 (34)	918 (25)	926 (29)	726 (18)
	Serious	435 (7)	164 (2)	270 (8)	93 (2)	165 (6)	71 (2)
Age (years)*	65–74	2409 (44)	4258 (63)	1257 (40)	2146 (63)	1152 (50)	2112 (63)
	75–84	2544 (39)	2653 (31)	1396 (40)	1218 (31)	1148 (37)	1435 (32)
	85–102	1636 (17)	713 (6)	806 (20)	246 (6)	830 (13)	467 (5)
Country of birth*	Sweden	5897 (85)	6904 (86)	3064 (85)	3240 (85)	2833 (85)	3664 (87)
	Other Nordic countries	396 (7)	442 (7)	238 (7)	254 (9)	158 (6)	188 (5)
	Rest of the world	296 (9)	278 (7)	157 (8)	116 (7)	139 (10)	162 (8)
Educational level*	College/ University	1405 (15)	2673 (26)	735 (15)	1389 (28)	670 (15)	1284 (24)
	High school	2923 (46)	3258 (47)	1590 (48)	1513 (47)	1333 (44)	1745 (47)
	Elementary school	2261 (39)	1693 (27)	1134 (37)	708 (25)	1127 (41)	985 (29)
Living with a spouse/partner*	Yes	3877 (59)	5335 (70)	1733 (50)	2249 (62)	2144 (70)	3086 (78)
	No	2712 (42)	2289 (30)	1726 (50)	1361 (38)	986 (30)	928 (22)
Participation in social activities in the last 12 months*	Yes	1996 (29)	3921 (51)	1070 (29)	1921 (52)	926 (29)	2000 (50)
	No	4593 (71)	3703 (49)	2389 (71)	1689 (48)	2204 (72)	2014 (50)
Having trust in other people*	Yes	5191 (77)	6417 (82)	2703 (76)	3040 (82)	2488 (77)	3377 (82)
	No	1398 (23)	1207 (18)	756 (24)	570 (18)	642 (23)	637 (18)
Having a friend to confide in*	Yes	5653 (85)	6871 (91)	3003 (87)	3310 (93)	2650 (84)	3561 (89)
	No	936 (15)	753 (10)	456 (13)	300 (7)	480 (17)	453 12)

The proportions are based on weighted data

Physically inactive = < 150 min/week, Physically active = ≥ 150 min/week

*P < 0.001

### Logistic regressions

Psychological distress was significantly associated with physical inactivity; the ORs for moderate and serious psychological distress were 1.85 (95% CI: 1.82–1.88) and 3.95 (95% CI: 3.80–4.10), respectively in those who were physically inactive compared with those being physically active (Table 3, crude model).

**Table 3:** Odds ratios and 95% CIs for total study population: psychological distress and physical inactivity

		Crude OR (95% CI)	Step 1 OR (95% CI)	Step 2 OR (95% CI)
Psychological distress*	No/low	Ref	Ref	Ref
	Moderate	1.85 (1.82–1.88)	1.75 (1.72–1.78)	1.58 (1.55–1.61)
	Serious	3.95 (3.80–4.10)	3.50 (3.37–3.64)	2.76 (2.65–2.87)
Living with a spouse/ Partner*	Yes			Ref
	No			1.26 (1.24–1.28)
Participation in social activities in the last 12 months*	Yes			Ref
	No			2.17 (2.13–2.20)
Having trust in other people*	Yes			Ref
	No			1.09 (1.07–1.11)
Having a friend to confide in*	Yes			Ref
	No			1.16 (1.13–1.19)
Nagelkerke		0.041	0.118	0.160

The analysis are conducted with weighted data

Adjustments are made in two different steps. Step 1 was adjusted for age, country of birth and educational level. Step 2 was adjusted for age, country of birth, educational level, living with a spouse/partner, participation in social activities in the last 12 months, having trust in other people and having a friend to confide in

*P < 0.001

In step 1, adjustments were made for background covariates (age, country of birth, level of education), and all included background covariates were significant (*p* < 0.001) in this model. In the fully adjusted model, step 2, the psychosocial covariates were included (living with a spouse/partner, participating in social activities during the last 12 months, having trust in other people, having a friend to confide in) and the ORs for moderate and serious psychological distress were 1.58 (95% CI: 1.55–1.61) and 2.76 (95% CI: 2.65–2.87), respectively. All included covariates were statistically significant (*p* < 0.001). The following were all associated with physical inactivity, not living with spouse/partner (OR = 1.26; 95% CI 1.24–1.28), not having participated in social activities during the last 12 months (OR = 2.17; 95% CI: 2.13–2.20), not having trust in other people (OR = 1.09; 95% CI: 1.07–1.11) and not having a friend to confide in (OR = 1.16; 95% CI: 1.13–1.19).

The Nagelkerke value increased from 0.041 in the crude model to 0.160 in the fully adjusted model in step 2.

### Stratified analysis for women and men

Since both psychological distress and physical inactivity were statistically different between women and men logistic regression analyses stratified by sex were performed.

In the crude model in Table 4, the ORs for the association between moderate psychological distress and physical inactivity were 1.70 (95% CI: 1.66–1.74) among women and 1.96 (95% CI: 1.91–2.01) among men. For the association between serious psychological distress and physical inactivity, the ORs were 4.18 (95% CI: 3.97–4.40) among women and 3.42 (95% CI: 3.23–3.62) among men. In step 1, after adjusting for age, country of birth, and level of education, all included variables were significant (*p* < 0.001) among women and men. In the fully adjusted model, step 2, living with a spouse/partner, participation in social activities in the last 12 months, having trust in other people and having a friend to confide in were included. The ORs for the association between moderate psychological distress and physical inactivity were 1.46 (95% CI: 1.43–1.50) among women and 1.70 (95% CI: 1.65–1.74) among men. Further, the ORs for the association between serious psychological distress and physical inactivity were 2.87 (95% CI: 2.72–3.04) among women and 2.43 (95% CI: 2.28–2.58) among men. All ORs were statistically significant (*p* < 0.001). Not living with a spouse/partner was associated with physical inactivity among women OR = 1.17 (95% CI: 1.14–1.20) and among men OR = 1.23 (95% CI: 1.20–1.26). An association was also seen between physical inactivity and not having participated in social activities during the last 12 months among both women, OR = 2.19 (95% CI: 2.15–2.24) and men OR = 2.13 (95% CI: 2.08–2.18). Not having trust in other people was also significantly associated with physical inactivity, OR = 1.10 (95% CI: 1.08–1.13) among women and OR = 1.06 (95% CI: 1.04–1.09) among men. Not having a friend to confide in was also significantly associated with physical inactivity, OR = 1.27 (95% CI: 1.23–1.32) among women and OR = 1.13 (95% CI: 1.09–1.17) among men.

**Table 4:** Odds ratios and 95% CIs for psychological distress and physical inactivity, by sex

		Women	Men	Women	Men	Women	Men
		Crude OR (95% CI)	Crude OR (95% CI)	Step 1 OR (95% CI)	Step 1 OR (95% CI)	Step 2 OR (95% CI)	Step 2 OR (95% CI)
Psychological distress*	*No/low	Ref	Ref	Ref	Ref	Ref	Ref
	Moderate	1.70 (1.66–1.74)	1.96 (1.91–2.01)	1.61 (1.58–1.65)	1.88 (1.84–1.93)	1.46 (1.43–1.50)	1.70 (1.65–1.74)
	Serious	4.18 (3.97–4.40)	3.42 (3.23–3.62)	3.75 (3.56–3.95)	3.00 (2.83–3.18)	2.87 (2.72–3.04)	2.43 (2.28–2.58)
Living with a spouse/partner*	Yes					Ref	Ref
	No					1.17 (1.14–1.20)	1.23 (1.20–1.26)
Participation in social activities in the last 12 months*	Yes					Ref	Ref
	No					2.19 (2.15–2.24)	2.13 (2.08–2.18)
Having trust in other people*	Yes					Ref	Ref
	No					1.10 (1.08–1.13)	1.06 (1.04–1.09)
Having a friend to confide in*	Yes					Ref	Ref
	No					1.27 (1.23–1.32)	1.13 (1.09–1.17)
Nagelkerke		0.041	0.038	0.146	0.089	0.186	0.130

The analysis are conducted with weighted data

Adjustments are made in two different steps. Step 1 was adjusted for age, country of birth, and educational level. Step 2 was adjusted for age, country of birth, educational level, living with a spouse/partner, participation in social activities in the last 12 months, having trust in other people and having a friend to confide in

*P < 0.001

Among women, the Nagelkerke value increased from 0.041 in the crude model to 0.186 in the fully adjusted model in step 2. The corresponding range among men was 0.038–0.130.

## Discussion

This study aimed to investigate the association between psychological distress and physical inactivity in adults aged ≥ 65 years and the role of psychosocial factors. Furthermore, this study aimed to explore if there were sex-based differences.

The older adults in our study who were physically inactive and reported a higher level of psychological distress, were older, had lower levels of education, lower trust in people, had fewer friends to confide in, lived to a lower extent with a spouse/partner and participated in social activities less often than those who were physically active.

In this study, a statistically significant association was found between nonparticipation in social activities and physical inactivity in both women and men. Similar results were confirmed in previous research of older adults with anxiety, in which low participation in social activities was significantly associated with physical inactivity [35]. Being part of social activities gives an opportunity for the individual to receive both social and emotional support, and research has shown that social support is essential for many individuals´ mental health [36]. Social support has been described as engaging in enjoyable activities with others. Emotional support involves having someone to engage with regarding talking and listening. A significant relationship was found at higher levels of distress following higher positive social interaction, however, the reciprocal relationship was not observed. Furthermore, higher levels of distress are a significant precursor to receiving more social support, suggesting that older adults may seek support to cope with the situation [36]. Also, sharing one´s innermost feelings offers emotional support and the presence of a confidant, both of which play a crucial role in mental health [37]. This is in line with the results of our study that showed not to have a friend to confide in, had a significant association with physical inactivity. Confiding in someone can differ between women and men, which was evident among older adults, where women sought support from friends to discuss their problems to a greater extent than men [37].

In addition to social support and having someone to confide in, older adults can also benefit from various interventions that improve their mental health. In a previous study of older adults at risk of depression or with subclinical depressive symptoms, psychosocial interventions that reinforced psychological or social factors had a positive effect [38]. Psychosocial interventions include physical exercise, reminiscence, social activities, skill training, and multicomponent interventions. The results showed statistically significant improvements in mental health and quality of life with the prevention of depressive symptoms [38].

Addressing social factors, social prescribing is increasingly recognized as an alternative intervention for older adults, originating in the United Kingdom and adopted internationally, particularly in high-income countries. This approach involves connecting socially isolated patients to suitable community activities and resources. Previous research has demonstrated that social prescribing positively affects loneliness, overall health, and well-being in older adults [39, 40].

Our study revealed that 45% of the 14,213 older adults were physically inactive, and 30% reported experiencing psychological distress. Additionally, a significant association was observed between psychological distress and physical inactivity. Our results are in agreement with those of previous studies showing that women are more physically inactive [1, 6]. However, the results of other studies do not support these findings, as they found older women and men to be equally physically inactive [3], and older men were more physically inactive than older women [4]. Additionally, the results in our study seem consistent with other research showing that women experience higher levels of psychological distress than men [13, 14]. Low physical activity among older adults with mental illness is also well known [6–8], as demonstrated in a previous study of older adults with anxiety, in which almost half (45%) did not meet the global recommendations for physical activity [35].

Furthermore, our results are consistent with those of another Swedish cross-sectional study involving older adults that explored the association between participation in leisure activities and psychological distress [41]. The findings revealed that women experienced significantly higher levels of psychological distress than men. Additionally, depression was strongly and negatively associated with leisure activities, with this association being even stronger in women than in men [41].

Psychological distress refers to normal reactions to stressful situations in life and lasts for a limited time [9–11]. Previous research showed that older adults described psychological distress as feelings of higher level of stress or feeling low [37]. These feelings were due to losses, such as the death of their partner or close friends, or deteriorating physical health, resulting in impaired mobility [37], which can increase individuals vulnerability to loneliness and social isolation [42]. Older adults has reported that their life experiences often enabled them to manage their psychological distress on their own, however if distress persists, they are more likely to seek help from primary care centers, especially if they trust their general practitioner. However, the stigma surrounding mental health issues can prevent some older adults from seeking the help they need [37].

Aging is commonly associated with an increased risk of physical and mental illnesse, which reduces the possibility of being physically active. A previous study on older adults reported that the probability of being physically active decreased by 7% for every year of individual age after the age of 75, and for each chronic disease, the likelihood of engaging in physical activity decreased by 8% [43]. Furthermore, aging is known to affect physical activity levels, implying that many older adults are physically inactive or have reduced physical activity levels [6, 28].

In primary care, creating prerequisites and encouraging older adults to be physically active is of great importance. To achieve this, it is essential to have knowledge of the factors that both enable and impede physical activity. Previous research has identified several key factors that influence physical activity among older adults [44]. Positive determinants included social support, being male, and living in a highly walkable area. In contrast, loneliness is a significant negative determinant [44]. The perception of physical activity among older adults in a previous study varied; some believed it to be unnecessary or even harmful, whereas others recognized its benefits [45]. Despite their knowledge of the benefits, older adults experience several barriers such as physical limitations, social influences, competing priorities, motivation, beliefs, and access difficulties. The results suggest the need to inform older adults about the positive and minor negative effects of engaging in physical activity, as well as to be aware of other individual factors that influence their decision to be physically active [45].

Based on the results of our study, it is of utmost importance not only to investigate the physical activity levels of older adults but also to consider their participation in social activities. To reduce the risk of psychological distress and enable a higher degree of physical activity in older women and men, our results imply that it is important to recommend and refer older adults to community group activities and other facilities that cater to older adults. Participating in regular social and physical group activities can provide an opportunity to reduce feelings of loneliness, offer emotional support, and have a positive impact on both mental and physical health.

### Strengths and limitations

One strength of this study is the large study population of 14,213 older adults, and the high response rate in LH 2022 (61%). The older adults were also those with the highest response rate compared to 45% for LH 2022 in total.

Furthermore, using a large population-based random sample from the total population of older adults, together with calibration weight allow the findings to be generalized to the population of Mid-Sweden and with some caution to other similar populations. The educational distribution among study participants was similar to that of the Swedish population, supporting the generalizability of the findings. The observed age-related differences in education levels (53% low education among 85–102-year-olds versus 24% among 65–74-year-olds) are consistent with national statistics in Sweden [29].

The data also allowed for subgroup analysis by sex to be performed.

For this study, specific questions related to psychosocial factors were chosen because we hypothesized that these variables might influence the association between psychological distress and physical inactivity.

Although this study has several strengths, some limitations should be addressed, particularly regarding self-reporting of physical activity, which can be biased. A systematic review indicated that self-reported physical activity may be both underestimated and overestimated [46]. Nevertheless, we used information from two different validated and recommended questions on self-reports of time spent in daily activities as well as physical exercise [30], to assess compliance with the recommended level of physical activity of ≥ 150- min/week by WHO [6].

Psychological distress was measured using the K6 scale, a tool widely used in previous research [31]. Previous studies have shown that it is sex-neutral [47] and that higher psychological distress levels in women than in men may indicate a true sex difference [33, 47]. However, its psychometric properties have not been validated in older adults in Sweden.

Moreover, 1,929 people were excluded as they lacked information on psychological distress, physical inactivity and psychosocial covariates. Psychosocial covariates and questions on psychological distress may be sensitive, and our results might therefore be underestimated, particularly for the oldest persons. Although the sex distribution was similar, the excluded individuals were older and had lower education levels, which may limit the generalizability of our findings to more socioeconomically disadvantaged or older population.

Another limitation is the cross-sectional design of this study, as it does not allow for elaboration on any direction of causality, nevertheless a robust association between psychological distress and physical inactivity was observed.

## Conclusions

Psychological distress is significantly associated with physical inactivity among older adults. Participation in social activities was identified as an essential factor in addressing physical inactivity. Social connections and physical inactivity are important factors to consider when supporting older adults’ mental health. Public health interventions should promote and raise awareness of physical and mental health as well as the social dimensions of aging, while also considering age and sex-based differences.

## Data Availability

The datasets generated and/or analysed during the current study are not publicly available due to confidentiality and regulations under the Swedish law (the Public and Privacy Act 2009: 400, Chap. 24, Sect. 8), but descriptive data in table form are available from the corresponding author on reasonable request.

## Abbreviations

WHO: World Health Organization
LH: Life and Health Survey
K6: Kessler-6
BMI: Body Mass Index
OR: Odds Ratio
CI: Confidence interval
